# Study on the *Nocardia seriolae* Antagonistic Bacterium in the Gut Microbiota of *Micropterus salmoides*

**DOI:** 10.3390/biology14091128

**Published:** 2025-08-26

**Authors:** Shiwei Xu, Qi Chen, Anna Liu, Shu Chen, Wanyi Chen, Shixin Qian, Lei Wang, Yihong Chen

**Affiliations:** 1 Institute of Modern Aquaculture Science and Engineering (IMASE)/Key Laboratory for Healthy and Safe Aquaculture, College of Life Science, South China Normal University, Guangzhou 510631, China; 2025010225@m.scnu.edu.cn (S.X.); dknehc12345@163.com (Q.C.); 2024023133@m.scnu.edu.cn (A.L.); 2024023128@m.scnu.edu.cn (S.C.); 2023022922@m.scnu.edu.cn (W.C.); qianshixin21@163.com (S.Q.); 2 Southern Marine Science and Engineering Guangdong Laboratory (Zhuhai), Zhuhai 519000, China

**Keywords:** *N. seriolae*, *M. salmoides*, *B. amyloliquefaciens*, nocardiosis, probiotics

## Abstract

The present study investigates the gut microbiota dynamics of *Micropterus salmoides* during *Nocardia seriolae* infection and identifies potential probiotic candidates for disease control in aquaculture. We artificially infected *M. salmoides* with *N. seriolae* and analyzed the gut microbiota of dying fish, asymptomatic fish during the peak mortality period, and healthy fish. The results showed that *N. seriolae* infection significantly altered the gut microbiota diversity. Based on analysis of gut microorganisms, we isolated and identified an *N. seriolae*-resistant *Bacillus* from the gut of *M. salmoides*, which was named *B. amyloliquefaciens* MS05 (BaMS05). BaMS05 demonstrated significant antibacterial activity against *N. seriolae* and other common pathogens of *M. salmoides* both in vitro and in vivo. Additionally, BaMS05 could colonize the gut of *M. salmoides* for at least five days and exhibited a protective effect against *N. seriolae* infection, reducing cumulative mortality by 40% at the fifth day. This study provides insights into the role of gut microbiota in disease resistance and highlights BaMS05 as a potential probiotic for preventing and controlling *N. seriolae* infections in aquaculture. Further research will focus on optimizing the application of BaMS05 and verifying its biosafety for practical use in *M. salmoides* farming.

## Simple Summary

*Nocardia*, as the causative agent of nocardiosis in fish, causes high morbidity and mortality rates. In this study, *Micropterus salmoides* were artificially infected with *Nocardia seriolae* to investigate the dynamic changes in the intestinal microbiota during infection and screened for a potential probiotic for aquaculture disease control, named BaMS05. This strain demonstrated significant inhibitory effects against common pathogens in both in vivo and in vitro experiments, along with the ability to colonize the intestinal tract. These findings provide both theoretical and experimental support for the prevention and treatment of nocardiosis.

## 1. Introduction

*Micropterus salmoides* (*M. salmoides*), commonly known as the largemouth bass, is a freshwater species indigenous to North America, classified within the family Percoidei, order Perciformes, and genus *Micropterus*. Recognized as a significant commercial freshwater species, *M. salmoides* is renowned for its vigorous feeding behavior, exceptional culinary qualities, and popularity in competitive angling, establishing it as a prominent freshwater fish in North America and various global regions [1]. The optimal thermal range for the growth of *M. salmoides* is approximately 25 °C, while temperatures exceeding 30 °C can adversely affect its growth performance. This species was introduced to Guangdong Province, China, in 1983, where it has subsequently emerged as a key species in Chinese aquaculture [2].

*M. salmoides* exhibits stringent environmental requirements in aquaculture, which can significantly influence its growth, development, and overall health. Inadequate farming conditions may compromise the immune function of *M. salmoides*, rendering it vulnerable to a range of diseases and potentially leading to mass mortality events. Recent studies indicate that various viral pathogens have inflicted substantial economic damage on the *M. salmoides* sector in China, with notable viral infections such as largemouth bass virus (LMBV), largemouth bass birnavirus (LBBV), and viral hemorrhagic septicemia virus (VHSV). Concurrently, bacterial infections remain a primary challenge hindering the sustainable advancement of *M. salmoides* aquaculture. The transmission of bacterial pathogens occurs through contaminated water, feed, equipment, and decaying organisms, facilitating rapid spread within aquaculture environments and complicating prevention and control efforts. Notable bacterial pathogens affecting *M. salmoides* farming include *Nocardia seriolae* (*N. seriolae*), *Aeromonas hydrophila*, *Aeromonas wiedemannii*, *Vibrio parahaemolyticus*, *Edwardsiella piscicida*, and *Francisella orientalis* [3,4,5,6,7]. Due to declining antibiotic use in aquaculture, disease prevention now relies on strategies like lower stocking density, water quality management, and probiotics. While probiotics are gaining attention as an alternative to antibiotics in fish farming, their widespread adoption is limited by the lack of efficient and sustainable aquaculture-specific strains.

In recent years, aquaculture of *M. salmoides* has faced significant challenges due to *Nocardia* infections, which pose a serious threat to their survival rates. The Nocardia species responsible for nocardiosis infections in farmed fish include *N. asteroides*, *N. salmonicida*, *N. seriolae*, and *N. brasiliensis* [8,9,10]. *Nocardia* typically induces visceral white spot disease, evidenced by external hemorrhaging, subcutaneous abscess formation, and the presence of white nodules in the kidneys and spleen [11,12]. The bacterium spreads hematogenously (via the bloodstream), leading to the formation of hyphae and granulomatous nodules in multiple organs. Due to its destructive effects on immune organs and the lack of targeted therapeutic interventions, treatment options remain complex. Standard therapeutic approaches include continuous disinfection using iodine or chlorine, administration of oral antibiotics (such as enrofloxacin and amoxicillin), and the use of traditional Chinese medicinal practices combined with vitamin K. The limited efficacy of these treatments is attributable to the poor penetration of therapeutic agents into the nodules. While vaccination could represent a viable and environmentally sustainable strategy, there are currently no approved vaccines for *Nocardia* in fish within China. Therefore, there is an urgent need for innovative strategies to mitigate *Nocardia* infections in aquaculture.

For aquaculture, the interaction between gut microbiota and fish diseases is crucial; a balanced gut microbiome boosts host immunity, while dysbiosis can lead to infections and illnesses. As reported, gut microbiota could contribute to disease resistance in fish through several mechanisms: (1) serving as a barrier against pathogens by competing for resources and producing antimicrobial substances [13]; (2) improving fish health via probiotics, prebiotics, or synbiotics [14,15]; and (3) influencing the immune response of the host through the gut–immune axis [16]. As a key indicator of fish health, gut microbiota present innovative approaches for sustainable aquaculture. Recent studies have identified effective methods for isolating pathogenic and antagonistic bacteria from fish intestines. For example, 65 *Bacillus subtilis* strains were isolated from the intestines of *Pelteobagrus fulvidraco*, with strain F14 demonstrating significant antibacterial activity against *A. hydrophila* through crude extract metabolism [17].

In this study, the intestinal microbiota community of *M. salmoides* artificially infected with *Nocardia* was analyzed, and a strain of *Bacillus amyloliquefaciens* MS05 (BaMS05) with potential resistance to *N. seriolae* was screened. Its biological characteristics were also explored in this study, providing a potential strategy for the biological control of *Nocardia* diseases in aquaculture.

## 2. Materials and Methods

### 2.1. Experimental Fish and N. seriolae

The *M. salmoides* used in the experiment were purchased from a farm in Foshan, Guangdong Province, weighing 20 ± 1 g. The experimental fish were confirmed to be free from parasitic and bacterial diseases such as *Nocardia* through PCR testing. Before the experiment began, the breeding system was thoroughly disinfected with potassium permanganate solution. During the temporary rearing period, the water temperature was strictly controlled at 25 ± 1 °C. The fish were fed twice a day, with the feeding amount being 3% of the fish’s body weight. The temporary rearing period lasted for two weeks, and the experimental fish were deprived of food for 24 h before the challenge experiment.

The strain of *N. seriolae* (NK0609) from the mandarin fish in our lab, stored at −80 °C, was inoculated onto brain–heart infusion (BHI) agar using streaking and inverted in a 28 °C incubator until single colonies formed. Subsequently, single colonies were picked from the agar and inoculated into BHI liquid medium, which was then cultured under conditions of 28 °C temperature and 150 rpm/min shaking for 4 d. After cultivation, the bacterial suspension was centrifuged at 4000 r/min for 15 min to collect the bacterial precipitate. The bacteria were resuspended in PBS buffer solution and washed twice. Another 4000 rpm/min centrifugation at 15 min collected the bacteria, after which an appropriate amount of PBS was added, and the bacteria were homogenized using a glass homogenizer to prepare a bacterial suspension. The concentration of the bacterial suspension was determined by plate colony count to be 4.6 × 10^8^ CFU/mL. Subsequently, 100 μL of the bacterial suspension was serially diluted with PBS at dilution factors of 10^−1^ or 10^−2^ for use in challenge experiments.

### 2.2. Detection of Pathogenic Ability of N. seriolae

**Figure 1:**
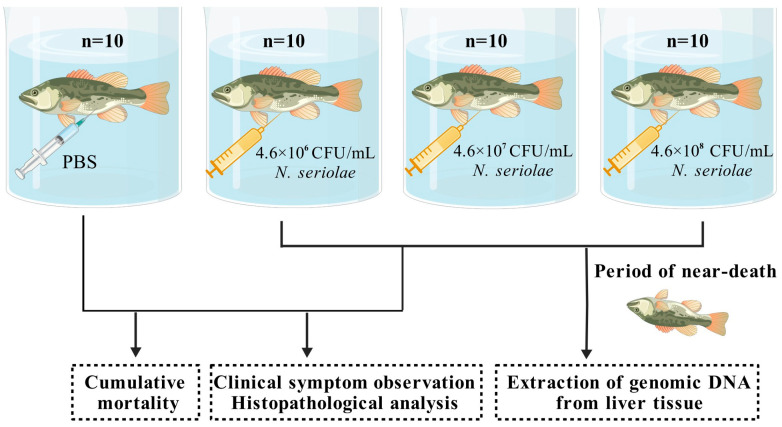
Experimental design diagram.

To evaluate the pathogenicity of the *Nocardia* strain in *M. salmoides*, a challenge test was conducted. Healthy, size-matched fish were randomly divided into control and experimental groups and placed in independent recirculating water aquaculture systems. The control group (*n* = 10) was injected with 100 μL of PBS. Three experimental groups (*n* = 10 in each group) were injected with *Nocardia* suspensions at concentrations of 4.6 × 10^6^, 4.6 × 10^7^, and 4.6 × 10^8^ CFU/mL. Following challenge, fish were monitored continuously. Moribund individuals from experimental groups were randomly selected, anesthetized and dissected for clinical symptom observation and histopathological analysis. Additionally, from the experimental group and the control group, moribund and healthy fish were randomly selected and dissections were performed to collect liver samples (Figure 1). Genomic DNA was extracted from the liver using the MiniBEST Universal Genomic DNA Extraction Kit Ver.5.0 (Takara, Kyoto, Japan). Detection of *Nocardia* in *M. salmoides* was performed using specific primers and PCR (Table 1), following the method outlined by Chen et al. [18]. The PCR reaction system was set to a final volume of 50 μL: primers (10 μM) 1 μL each, 2 × Taq Plus Master Mix II (Vazyme, Nanjing, China) 25 μL, DNA template 2 μL, with sterile distilled water for volume completion. The optimized thermal cycling parameters were 95 °C for 3 min to activate the polymerase, followed by 32 cycles of 95 °C for 30 s, 55 °C for 30 s, and 72 °C for 36 s, and the final extension step at 72 °C for 5 min.

### 2.3. LD50 Determination of N. seriolae Infection in M. salmoides

After different doses of *N. seriolae* injection in *M. salmoides*, continuous observation was carried out for 14 d and mortality was calculated by Kaplan–Meier survival curve analysis. The 14 d LD_50_ of *M. salmoides* was calculated by the Reed–Muench method, and its calculation formula is as follows: lgLD_50_ = r (50 − b) + *n*, r = (m − n)/(a − b), where r is the increase in dose logarithm for every 1 percentage point increase in mortality; a is the cumulative mortality rate greater than 50%; m is the logarithmic dose corresponding to a cumulative mortality greater than 50%; b is the cumulative mortality rate less than 50%; and n is the logarithm of the dose corresponding to the cumulative mortality rate less than 50%.

### 2.4. Preparation of Samples for Intestinal Flora Analysis

**Figure 2:**
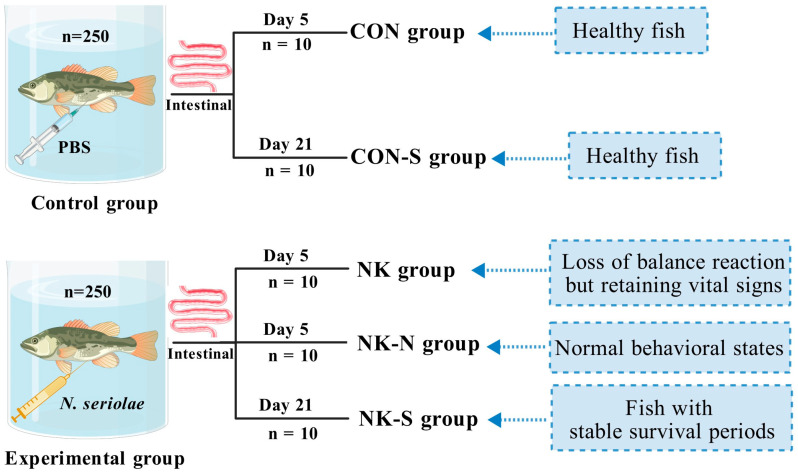
Experimental design and sample collection process.

In order to obtain samples for gut microbiota analysis, another challenge experiment was conducted. A total of 500 *M. salmoides* with consistent body length (8 ± 1 cm) and weight (20 ± 1 g) were selected randomly and divided into two groups (250 fish per group). They were then placed in independent recirculating water aquaculture systems (Figure 2). The experimental group was intraperitoneally injected with 100 μL of a 5 × 10^7^ CFU/mL *N. seriolae* suspension, while the control group was injected with the same volume of PBS. At the peak of infection and death (on the 5th day after injection), 10 fish exhibiting near-death states (loss of balance reaction but retaining vital signs) were randomly selected from the experimental group (NK group), 10 fish were selected from the experimental group with normal behavioral states (NK-N group), and 10 healthy fish were randomly selected from the control group (CON group). The external fish surface was disinfected by wiping with 75% ethanol. Using sterile dissecting scissors, an arc-shaped incision was made anterior to the anal vent. The abdominal cavity was opened, the intestinal tract was extracted, and associated adipose tissue was meticulously removed using forceps. The intestinal surface was cleaned by wiping with a 75% ethanol-saturated sterile swab and the sample was placed in a 1.5 mL EP tube. All samples were immediately subjected to DNA extraction.

During the stable infection period (on the 21st day after injection, no more deaths occurred in the experimental fish), 10 fish from the experimental group (NK-S group) and 10 fish from the control group (CON-S group) were randomly selected. The external fish surface was disinfected by wiping with 75% ethanol. Using sterile dissecting scissors, an arc-shaped incision was made anterior to the anal vent. The abdominal cavity was opened, the intestinal tract was extracted, and associated adipose tissue was meticulously removed using forceps. The intestinal surface was cleaned by wiping with a 75% ethanol-saturated sterile swab and the samples were placed in a 1.5 mL EP tube. All samples were immediately subjected to DNA extraction (Figure 2).

### 2.5. 16S rRNA Amplification and Sequencing and Data Processing and Analysis

Genomic DNA was extracted from *M. salmoides* intestinal samples using the MiniBEST Universal Genomic DNA Extraction Kit Ver.5.0 (Takara, Kyoto, Japan). DNA integrity and concentration were verified through agarose gel electrophoresis and NanoDrop spectrophotometry to ensure suitability for subsequent amplification procedures. The DNA amplification and sequencing were carried out by Guangzhou Aiji Biotechnology Co., Ltd. (Guangzhou, China). The process is as follows: Specific primers were designed and synthesized for the 16S rDNA V3-V4 region for targeted amplification. The specific primers were 341F: 5′-CCTACGGGNGGCWGCAG-3′; 805R: 5′-GACTACHVGGGTATCTAATCC-3′. PCR products were purified using AMPure XT beads (Beckman Coulter Genomics, Danvers, MA, USA). The purified products were quantified using a Qubit 3.0 Fluorometer (Thermo Fisher Scientific, Waltham, MA, USA). The amplification success and specificity were verified by 2% agarose gel electrophoresis, and the electrophoresis bands were recovered using the AMPure XT beads recovery kit. The quality of the recovered products was evaluated using library quantification kits for Agilent Bioanalyzer 2100 (Agilent, Santa Clara, CA, USA) and Illumina (Kapa Biosciences, Woburn, MA, USA) to ensure that the library concentration was no less than 2 nM. Qualified libraries were diluted according to the required sequencing volume, mixed proportionally, and subjected to NaOH denaturation into single strands for sequencing; dual-end sequencing was performed using an NovaSeq 6000 sequencer. For the paired-end data obtained from sequencing, it is first necessary to split the sample data based on barcode information and remove adapters and barcode sequences. Following the procedures of data splicing and filtering, ASV (feature) sequences and abundance tables were acquired while excluding singleton ASVs. Alpha and beta diversity analyses were performed utilizing the resulting ASV (feature) sequences and abundance tables. Using the ASV (feature) sequence file, species were annotated with the SILVA database referencing the NT-16S database, and a statistical analysis of the species abundance across different samples was conducted based on the ASV (feature) abundance table. Finally, differential analysis was carried out between comparison groups based on the statistical data of species abundance.

### 2.6. Bacillus Spores Isolated from the Gut of M. salmoides

From the NK-S group, *M. salmoides* with no external damage and in good health were randomly selected. They were anesthetized with 20 mg/L MS-222, then cleaned with 75% alcohol. The intestines were taken out in an aseptic environment on an ultra-clean workbench and placed in a disposable petri dish. The surface of the gut was rinsed three times with cool PBS to ensure that the blood and fat were removed, and then placed in 2 mL PE tube with PBS buffer for homogenization treatment. The homogenized tissue was placed in a water bath at 80 °C for 20 min, coated on Luria-Bertani (LB) agar plate, and the culture inverted in a 37 °C incubator for 24~48 h; after colony growth, different morphological single colonies were selected according to the morphology, size and color of the colony with a sterilizing ring cultured again at 37 °C for 24~48 h, then purified three times to obtain multiple pure strains.

The *N. seriolae* (NK0609) and the isolated pure strains were cultured until the optical density (OD600) reached 1. After centrifugation and washing, the bacteria were resuspended in PBS to obtain the bacterial suspension. The *N. seriolae* were spread on the surface of a BHI agar medium. After the bacterial suspension was completely absorbed, a sterile puncher was used to make three holes at equal distances on the agar medium, and 50 μL of the pure strain suspension was added to each hole. The plates were incubated at 37 °C for 4 d. After the cultivation, the size of the inhibition zone was observed, and the strains with significant inhibitory effects on *Nocardia* were selected as candidate strains for further study.

### 2.7. Morphological, Biochemical and Molecular Biological Identification of Candidate Bacteria

The screened candidate strain was inoculated into LB liquid medium for cultivation. After 24 h, a sample of the bacterial culture was plated onto LB agar and incubated for 12 to 24 h to assess colony morphology. Concurrently, the bacterial solution was appropriately diluted and subjected to Gram staining using the Biosharp Gram Staining Kit, followed by microscopic examination.

The screened candidate strain was sent to Zhongke Testing Technology Service (Guangzhou) Co., Ltd. (Guangzhou, China). for physiological and biochemical profiling, assessing its spore formation capacity, NaCl tolerance, starch hydrolysis capability, fermentation of D-xylose and L-arabinose, volatile fatty acid (VFA) production, utilization of citrate, fermentation of D-mannitol, and gelatin liquefaction ability, among other characteristics.

The screened candidate strain was inoculated into LB liquid medium and cultured in a shaker at 37 °C for 4 to 8 h until turbid growth was achieved. PCR amplification of the bacterial DNA was performed, targeting the 16S rRNA gene using universal bacterial primers: 341F (CCTACGGGNGGCWGCAG) and 805R (GACTACHVGGGTATCTAATCC). The amplified products were sent to Guangzhou Aiji Biotechnology Co., Ltd. for sequencing. Subsequent homology analysis was conducted using the NCBI database to classify the species of the screened candidate strain based on the obtained gene sequences.

### 2.8. BaMS05 Growth Curve Determination

BaMS05 strain was inoculated into LB liquid medium and the culture shaken at 37 °C in a constant-temperature shaker (180 rpm) for 24 h to obtain seed solution. Then, the solution was inoculated at a ratio of 1% into 50 mL LB liquid medium and cultivation continued at 37 °C, 180 rpm. Samples were taken every 3 h in the first 30 h of cultivation, and every 6 h thereafter. The optical density of the bacterial solution was determined at 600 nm wavelength using a UV-Vis spectrophotometer, with time as the *x*-axis and OD600 value as the *y*-axis, to plot the growth curve of the strain.

### 2.9. Effects of Culture Parameters on BaMS05 Growth

Effect of temperature on the growth of BaMS05: the BaMS05 strain was inoculated in LB liquid medium with a 1% inoculum, and placed under 5 temperature gradients of 10 °C, 20 °C, 30 °C, 40 °C, and 50 °C. The culture was shaken at 180 rpm for 24 h. The LB liquid medium without the BaMS05 strain inoculation was set as the control group, with 3 biological replicates for each treatment. Samples were taken every 3 h to measure the OD600 value.

Impact of pH on the growth of BaMS05: A quantity of 50 mL of LB liquid medium was prepared in 200 mL Erlenmeyer flasks. The initial pH of the medium was adjusted using 1 mol/L HCl or 1 mol/L NaOH, with pH gradients set at 4, 5, 6, 7, 8, and 9. The culture medium was sterilized at 121 °C and 0.1 MPa, then cooled to room temperature before use. BaMS05 strain was inoculated with 1% inoculum, with 3 biological replicates for each treatment. All flasks were shaken at 30 °C and 180 rpm for 24 h, with samples taken every 3 h to measure the OD600 values.

The effect of NaCl concentration on the growth of BaMS05: A quantity of 50 mL LB liquid medium was prepared in 200 mL Erlenmeyer flasks, with NaCl concentrations set at 0.1%, 0.5%, 1%, 2%, 5%, 10%, and 20%. The BaMS05 strain was inoculated at 1% inoculum size for each treatment, with 3 biological replicates per treatment. All culture flasks were shaken at 30 °C and 180 rpm for 24 h, with samples taken every 3 h to measure the OD600 value.

### 2.10. Determination of the Antibacterial Activity of BaMS05

Strain BaMS05 and test pathogens (*N. seriolae*, *A. hydrophila*, *V. parahaemolyticus*, *V. alginolyticus*, and *S. agalactiae*) were individually cultured until the optical density (OD600) reached 1. After centrifugation and washing, 100 μL of the bacterial solution was extracted. *N. seriolae* and *S. agalactiae* were inoculated on BHI agar, and after complete absorption, a puncher was used to make three equidistant holes on the agar, adding 50 μL of BaMS05 bacterial fermentation liquid to each hole. In parallel, another set of BHI plates was prepared by spreading 100 μL of *N. seriolae* or *S. agalactiae* suspensions. After absorption, antibiotic discs containing gentamicin, streptomycin, penicillin, and a blank control disc were placed equidistantly as positive and negative controls. Additionally, after centrifugation and washing, 100 μL of *A. hydrophila*, *V. parahaemolyticus* and *V. alginolyticus* bacterial liquids were inoculated on LB agar, and the same procedures as mentioned above were followed.

### 2.11. Detection of Colonization Ability of BaMS05 in Gut of M. salmoides

We randomly selected 100 healthy *M. salmoides* with consistent body length (8 ± 1 cm) and weight (20 ± 1 g), and divided them into a control group (*n* = 50) and an experimental group (*n* = 50). All *M. salmoides* were fasted for 3 d before the experiment. The experimental group was fed feed containing 10^9^ CFU/g of BaMS05 spore bacteria twice, while the control group was fed sterile conventional feed twice, with a one-day interval between each feeding. Subsequently, all *M. salmoides* were fasted for 7 days. Sampling was done at 0 h of the second feeding, and at 1 h, 6 h, 1 d, 3 d, 5 d, and 7 d thereafter, with 5 *M. salmoides* being dissected at each time point. During the dissection, the intestines of the experimental *M. salmoides* were taken out, the intestinal contents were scraped off, the cleaned intestines were washed 3 times with sterile PBS, and the intestines were placed in a 2 mL centrifuge tube. A quantity of 1 mL of sterile PBS was added for homogenization, and the homogenate was heated at 80 °C for 20 min. The slurry was then spread on LB agar culture medium, with 100 μL on each plate, and incubated at 37 °C for 24 h to count the number of bacterial colonies.

### 2.12. Detection of the Protective Effect of BaMS05 on M. salmoides

BaMS05 and *N. seriolae* (NK0609) were cultured to 10^8^ CFU/mL. The experimental group was intraperitoneally injected with a mixture of 50 μL of BaMS05 + 50 μL of *N. seriolae*, while the control group was injected with 50 μL of BaMS05 + 50 μL of PBS, and 50 μL of *N. seriolae* + 50 μL of PBS buffer, respectively. The death toll of *M. salmoides* was observed and recorded continuously for 14 d.

### 2.13. Statistical Analysis

All data were subjected to three independent repeated experiments, and the statistical analysis of the data was conducted using GraphPad Prism 9.5 software. First, Shapiro–Wilk normality test and Levene’s homogeneity of variance test were conducted. Since the measured data met both the normal distribution and homogeneity of variance assumptions, Student’s *t*-test was used for pairwise comparisons between groups to analyze the species with differences. Additionally, the LEfSe method was used to analyze the characteristics of differentially abundant species between groups; the non-parametric Kruskal–Wallis (KW) sum-rank test was utilized to detect the differences in species abundance among different groups; then, the Wilcoxon rank-sum test was applied to verify the consistency of the differences in differential species among subgroups within different groups; finally, LDA discriminant analysis was employed to estimate the influence of these differential species on the distinction between groups. Statistical analysis results were considered statistically significant at *p* < 0.05.

## 3. Results

### 3.1. Infection of N. seriolae Resulted in Tissue Damage and Death in M. salmoides

**Figure 3:**
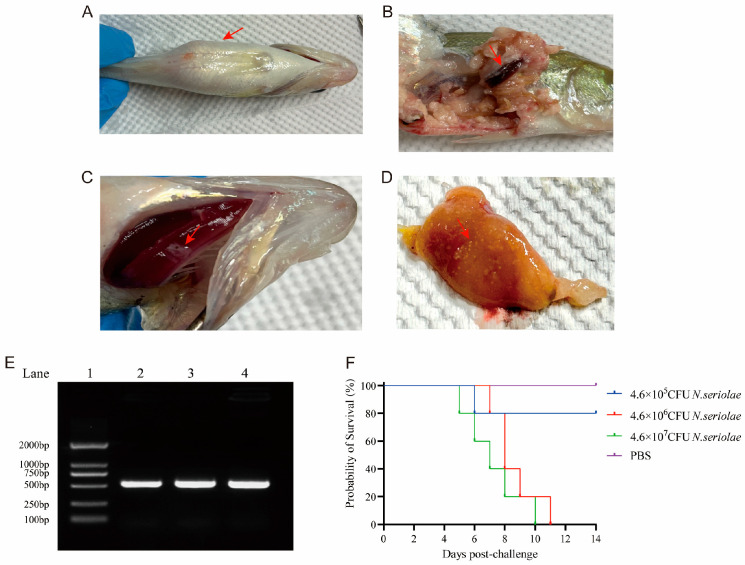
*Nocardia* infection in *M. salmoides*. (**A**) Abdomen. (**B**) Spleen. (**C**) Gill. (**D**) Liver. (The red arrow indicates symptoms of *Nocardia* infection.) (**E**) Partial sequence amplification of *M. salmoides* liver. (**F**) Dose-dependent survival curves of *M. salmoides* after *Nocardia* challenge.

After inoculation with *N. seriolae*, *M. salmoides* showed pathological changes, mainly characterized by delayed response, spinning at the bottom of the water, loss of body balance and swimming sideways, as well as decreased appetite. External clinical symptoms also included abdominal swelling, presence of ascites upon abdominal dissection, obvious white nodules on the gills, spleen, and liver surface, and liver surface showing nodules similar to a star-shaped appearance (Figure 3A–D). Specific PCR amplification of liver DNA extracted from diseased *M. salmoides* showed a single and clear band between 500~750 bp, indicating that *M. salmoides* successfully infected *N. seriolae* (Figure 3E). Mortality in *M. salmoides* infected with *N. seriolae* began at least 4 d after infection, and the mortality rate varied with different concentrations: fish in the 4.6 × 10^6^ CFU/mL and 4.6 × 10^7^ CFU/mL groups died within 14 d, with the latter group experiencing faster mortality compared to the former; while fish in the 4.6 × 10^5^ CFU/mL group showed 20% mortality in the early stages of the experiment, with no further mortality during the experiment period (Figure 3F). The 14 d LD_50_ value of *N. seriolae* strain NK0609 for *M. salmoides* was calculated to be 2.59 × 10^6^ CFU/fish.

### 3.2. The Sequencing Quality of Gut Microbiota in M. salmoides Is satisfactory 

**Figure 4:**
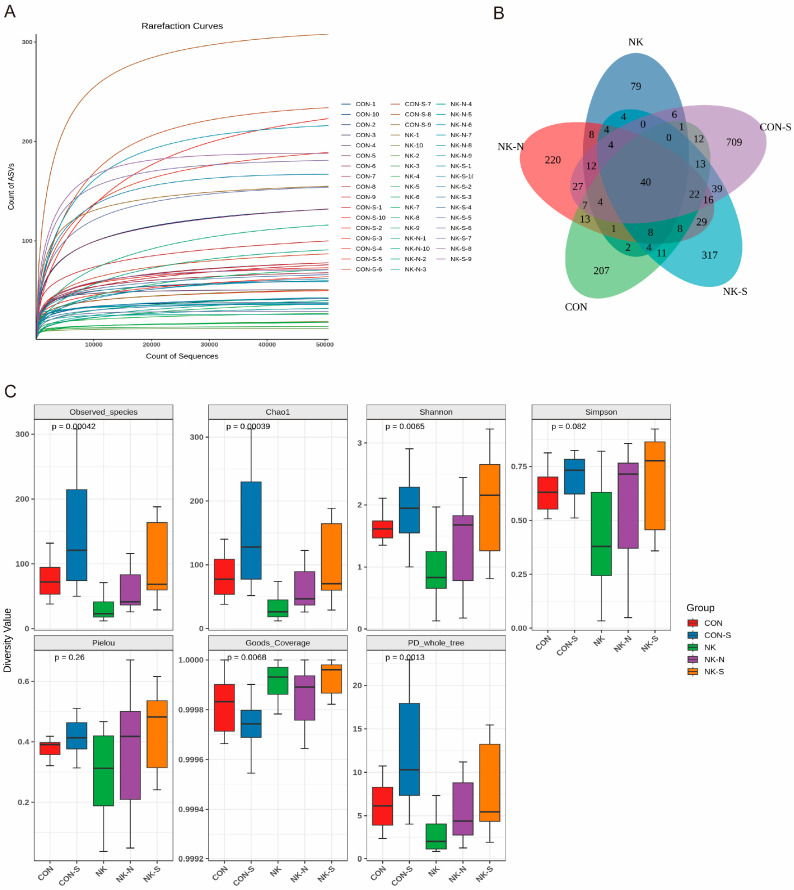
Gut microflora diversity in *M. salmoides* after *Nocardia* infection. (**A**) Rarefaction curve. The *x*-axis represents sequencing effort (number of reads), and the *y*-axis shows the cumulative number of observed ASVs at each sequencing depth. (**B**) Venn diagrams. (**C**) Boxplots showing alpha diversity across groups. The *x*-axis represents group labels, and the *y*-axis indicates the values of the alpha diversity index. *p*-values were calculated using the Wilcoxon rank-sum test.

Sequencing and quality control filtering were performed on all samples using the Illumina platform to ensure the quality of the data. The Q20 and Q30 values of each group reached high levels, indicating a high accuracy and reliability of the sequencing data. In addition, the quantity and quality of effective data in the control and infected groups met the requirements for subsequent genomic analysis (Table S1). Overall, the differences in clean data between the groups were small, indicating a stable sequencing and data processing process suitable for further genomic analysis and application. The Rarefaction Curve indicated (Figure 4A) that a large number of species were discovered in the gut of *M. salmoides* and did not significantly increase with the increase in sequencing quantity, suggesting that the sample sequences were sufficient for data analysis.

### 3.3. Differences in Gut Microbiota Diversity Exist Among the Experimental Groups

As shown in Figure 4B, Observed_species, Chao1, Shannon, Goods_Coverage, and PD_whole_tree showed significant differences, indicating that the sequencing depth was sufficient and the inoculation of *N. seriolae* altered the diversity of the intestinal microbiota of *M. salmoides* significantly. Under normal conditions (control group), there were significant differences in the intestinal microbiota diversity among *M. salmoides* individuals, similar to previous studies, indicating differences in gut microbial diversity among individuals. Differences also existed after inoculation with pathogenic bacteria, possibly due to differences in individual immune capabilities. Comparing the impact of *N. seriolae* infection on the α diversity of intestinal bacterial communities, the results showed the number of species in the gut microbiota: NK-S group > NK-N group > NK group. Further comparison of the Shannon diversity of intestinal bacterial communities at different treatments and time points showed that the Shannon diversity of the NK-N group was higher than the CON group, the Shannon diversity of the NK group was lower than the CON group, and the Shannon diversity of the NK-S group was higher than the CON-S group. The use of Usearch software 11.0.667 for clustering Reads at a 97.0% similarity level resulted in the identification of OTUs (Figure 4C), with 353 OTUs in the CON group, 177 OTUs in the NK group, 423 OTUs in the NK-N group, 912 OTUs in the CON-S group, and 519 OTUs in the NK-S group. Based on weighted PCA analysis, the injection of *N. seriolae* caused a change in the gut microbiota structure of *M. salmoides*, with the species composition of NK, NK-N, and NK-S being similar. The species composition at different time points also differed, with NK-S showing a slight downward shift on the *y*-axis compared to NK-N and NK, and CON-S showing a slight downward shift on the *y*-axis compared to the CON group (Figure S1).

### 3.4. The Effect of N. seriolae on the Gut Microbial Composition of M. salmoides

**Figure 5:**
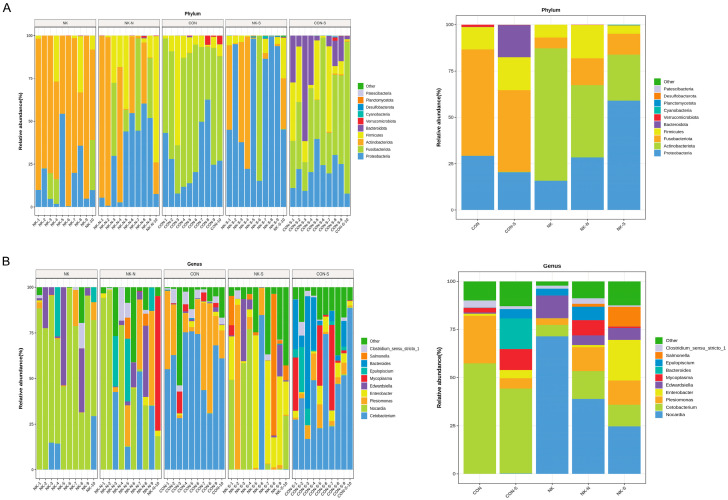
The relative abundance of gut microflora at the phylum and genus level. (**A**) Composition and relative abundance of microbial species in different individuals and groups at phylum level. (**B**) Composition and relative abundance of microbial species in different individuals and groups at genus level.

According to the species composition analysis, the dominant bacterial phyla in different treatments and periods were found. At the phylum level, the main dominant phyla in all treatment groups included the following: Patescibacteria, Desulfobacterota, Planctomycetota, Cyanobacteria, Verrucomicrobiota, Bacteroidota, Firmicutes, Fusobacteriota, Actinobacteriota, and Proteobacteria (Figure 5A). Specifically, the main dominant phyla in the CON group (relative abundance > 10%) were Fusobacteriota (57.35%) and Proteobacteria (29.26%). The main dominant phyla in the CON-S group were Fusobacteriota (44.09%), Proteobacteria (20.33%), Firmicutes (17.86%), and Bacteroidota (17.15%). In the NK group, the main dominant phyla were Actinobacteriota (71.483%) and Proteobacteria (15.80%). The main dominant phyla in the NK-N group were Actinobacteriota (38.99%), Proteobacteria (28.43%), Firmicutes (17.84%), and Fusobacteriota (14.55%). Lastly, the main dominant phyla in the NK-S group were Proteobacteria (59.05%), and Actinobacteriota (24.87%). Analyzing at the genus level, the main dominant genera in all treatment groups included the following: Clostridium sensu_stricto_1, Salmonella, Epulopiscium, Bacteroides, Mycoplasma, Edwardsiella, Enterobacter, Plesiomonas, Cetobacterium, and Nocardia (Figure 5B). Specifically, the main dominant genera in the CON group (relative abundance > 10%) were *Cetobacterium* (57.35%) and *Plesiomonas* (24.74%). In the CON-S group, the main dominant genera were *Cetobacterium* (44.094%), *Bacteroides* (15.93%), and *Mycoplasma* (10.86%). The main dominant genera in the NK group were *Nocardia* (71.48%) and *Edwardsiella* (11.82%). The main dominant genera in the NK-N group were *Nocardia* (38.92%), *Cetobacterium* (14.55%), and *Plesiomonas* (12.33%). Lastly, the main dominant genera in the NK-S group were *Nocardia* (24.745%), *Enterobacter* (21.052%), *Plesiomonas* (12.57%), *Cetobacterium* (11.18%), and *Salmonella* (10.22%).

LefSe analysis showed that in the NK-S group (Figure S2), various taxonomic groups such as Enterobacteriaceae and Gammaproteobacteria were significantly enriched. Among them, Verrucomicrobia, Curvibacter, and *Bacillus* were considered as potential beneficial bacteria and candidates for *Nocardia* antagonistic bacteria. In the NK-N group, the taxonomic group of Paraclostridium was highly enriched. In the NK group, the taxonomic groups of Corynebacteriales, Nocardiaceae, and Actinobacteria were significantly enriched. The dominant microbiota in the control group (CON group and CON-S group) was relatively diverse. In the CON group, the taxonomic groups of Pseudomonas, Bacillales, and Clostridiales were highly enriched; while in the CON-S group, the taxonomic groups of Bacilli and Bacteroidetes were significantly enriched.

**Figure 6:**
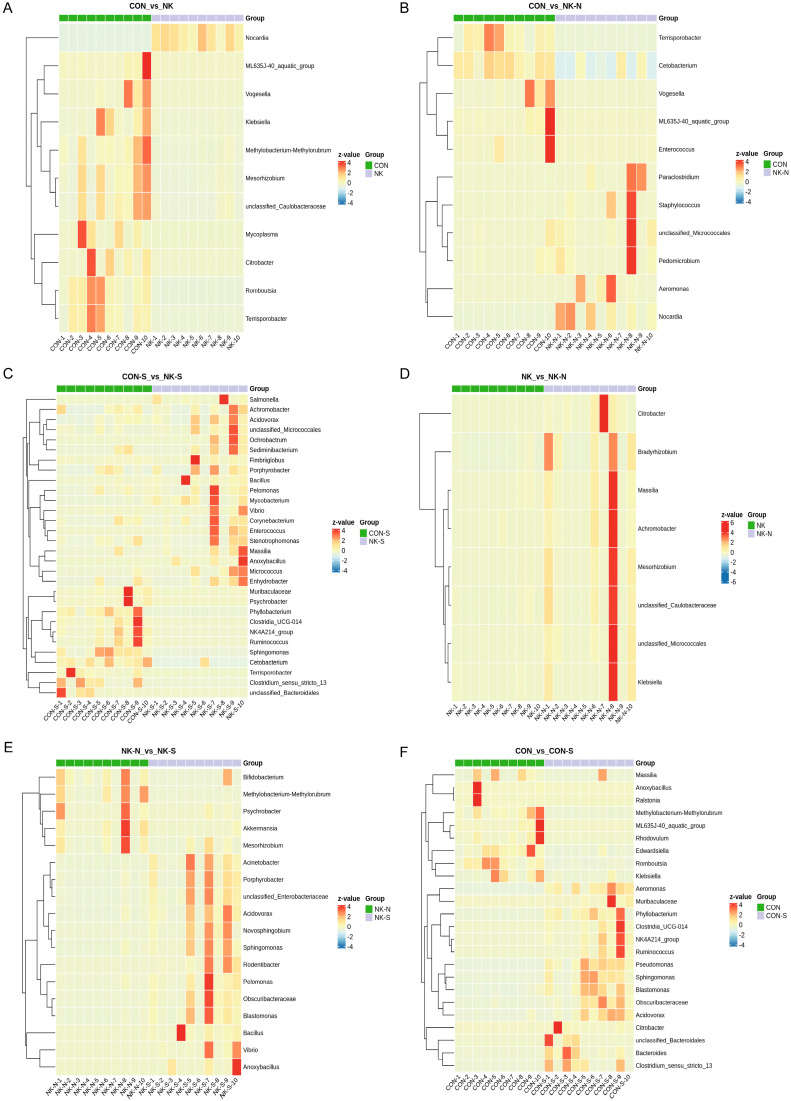
Microbial biomarker analysis in *M. salmoides* using metagenomeSeq. Pairwise between-group comparisons analyzed by metagenomeSeq. The heatmap displays significantly differential taxa (*p* < 0.05) with sample groups on the *x*-axis and taxonomic annotations on the *y*-axis. (**A**) Comparison between the CON group and the NK group. (**B**) Comparison between the CON group and the NK-N group. (**C**) Comparison between the CON-S group and the NK-S group. (**D**) Comparison between the NK group and the NK-N group. (**E**) Comparison between the NK-N group and the NK-S group. (**F**) Comparison between the CON group and the CON-S group.

When analyzing the samples using the metagenomeseq method, pairwise comparisons can avoid the impact of uneven sequencing depth. A zero-inflated Gaussian mixture model is also applied to address the effect of abundance differences caused by insufficient sampling. In this study, metagenomeseq was used to perform differential abundance analysis at the genus level. For the identified different genera (*p* < 0.05), the top 50 genera with the smallest *p*-values were selected, and a heatmap was generated based on their abundance. The results showed (Figure 6A,B) that compared to the CON group, the relative abundance of *N. seriolae* was significantly increased in the NK group, indicating successful colonization of *N. seriolae* in the gut of *M. salmoides*. The CON group had more different microbial communities compared to the NK group, possibly due to the significant impact of *N. seriolae* colonization on the gut microbial community structure, occupying an ecological niche in the gut. When comparing the CON-S group to the NK-S group, *Achromobacter*, *Acidovorax*, unclassified *Micrococcales*, *Ochrobactrum*, *Sediminibacterium*, *Fimbriiglobus*, *Porphyrobacter*, *Bacillus*, *Pelomonas*, *Mycobacterium*, *Vibrio*, *Corynebacterium*, *Enterococcus*, *Stenotrophomonas*, *Massilia*, *Anoxybacillus*, *Micrococcus*, and *Enhydrobacter* were significantly upregulated (Figure 6C,D). When comparing the NK-N group to the NK group, *Citrobacter*, *Bradyrhizobium*, *Massilia*, *Achromobacter*, *Mesorhizobium*, unclassified *Caulobacteraceae*, unclassified *Micrococcales*, and *Klebsiella* were significantly upregulated. The NK-S group showed significant upregulation of *Acinetobacter*, *Porphyrobacter*, unclassified *Enterobacteriaceae*, *Acidovorax*, *Novosphingobium*, *Sphingomonas*, *Rodentibacter*, *Pelomonas*, *Obscuribacteraceae*, *Blastomonas*, *Bacillus*, *Vibrio*, and *Anoxybacillus* compared to the NK-N group (Figure 6E,F).

**Figure 7:**
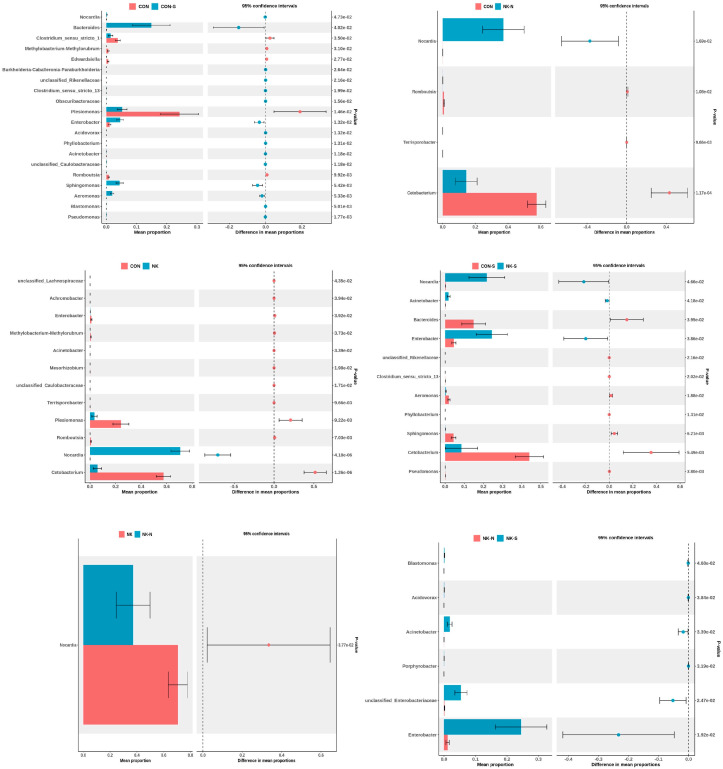
Pairwise group comparisons of the top 20 differentially abundant genera (STAMP plot representation). Species annotation information is displayed vertically, with the left bar showing the mean relative abundance of the differential species within each group, and the right bar chart shows the difference degree of the different species in two groups.

Using the independent two-tailed Student’s T-test to perform differential analysis of species abundance at the genus level, the identified differential genera (*p* < 0.05) were selected. The 20 genera with the smallest *p*-values were chosen for generating a STAMP plot (Figure 7). The results are as follows: compared to the CON group, the relative abundance of *Nocardia* in the NK group significantly increased; compared to the NK-N group and the CON group, the relative abundance of *Nocardia* in the NK-N group also significantly increased; compared to the CON-S group and the NK-S group, the relative abundance of *Nocardia* and *Enterobacter* in the NK-S group increased. In addition, there were many different genera between the CON group and the CON-S group, which may be related to individual differences in the composition and abundance of intestinal flora among different fish. Compared to the NK-N group, the relative abundance of *Nocardia* in the NK group was higher. Compared to the NK group, the NK-S group showed significant increases in the relative abundance of *Blastomonas*, *Acidovarax*, *Acinetobacter*, *Porphyrobacter*, unclassified *Enterobacteriaceae*, and *Enterobacter* genera.

### 3.5. Physiological Characteristics Analysis and Identification of BaMS05

**Figure 8:**
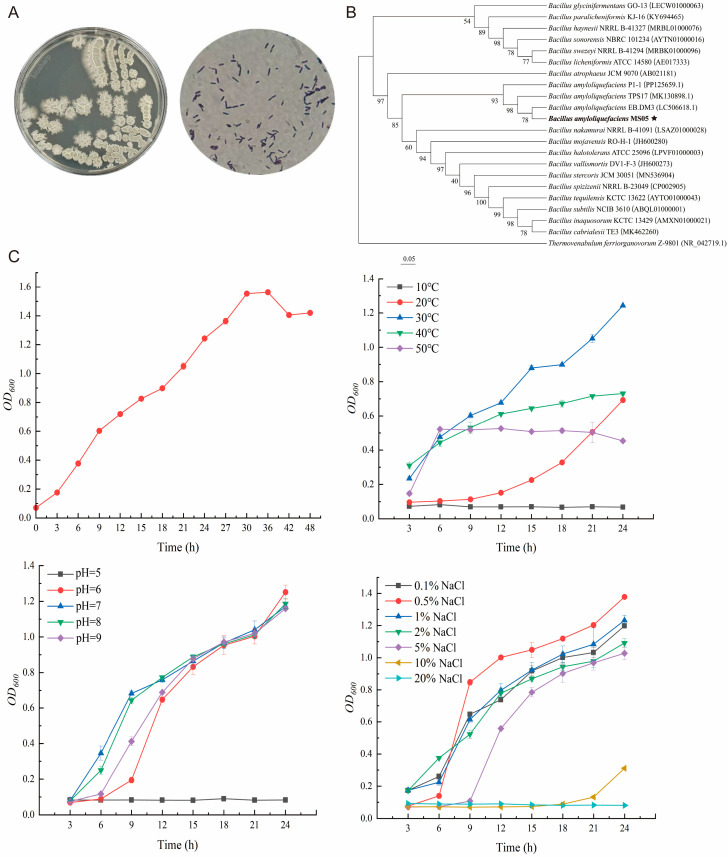
Morphological, biochemical and molecular biological identification of BaMS05. (**A**) Colony morphology and microscopic characterization. (**B**) Phylogenetic tree of partial 16S rRNA gene sequences of BaMS05. *Bacillus amyloliquefaciens* MS05 is marked with asterisks and bold font. (**C**) Growth kinetics of BaMS05: Standard cultivation curve and growth responses to varying conditions (temperature, pH, NaCl concentration).

In this study, 30 strains of *Bacillus* species were initially isolated from the intestine of *M. salmoides*, and BaMS05 was selected for antibacterial activity testing. Preliminary experimental results showed that BaMS05 could form obvious inhibition zones on a *Nocardia* agar plate, indicating its antibacterial activity. Therefore, this strain was selected for further research. On LB agar plate, BaMS05 colonies were dull, opaque, light yellow, and wrinkled; Gram staining was positive, with short rods that could form spores (Figure 8A). Physiological and biochemical identification results (Table 2) showed that BaMS05 could grow at pH 5.7 and in 7% NaCl, tested negative in the propionate and D-mannitol experiments, and tested positive in starch hydrolysis, gelatin liquefaction, D-xylose/L-arabinose/VP (Voges–Proskauer) test, citrate utilization, nitrate reduction, and citrate growth experiments. By comparing with the physiological and biochemical characteristics of other starch-hydrolyzing *Bacillus* strains [19] and through identification using the “Manual of Common Bacterial Systematic Identification” and morphological identification, this strain was tentatively identified as a starch-hydrolyzing *Bacillus*.

The obtained *Bacillus* spore 16S rRNA gene sequence was aligned with known sequences in the NCBI database, and a phylogenetic tree was constructed using MEGA 11 software. The Minimum Evolution method was used for the analysis, with P-distance method for constructing the phylogenetic tree. The results of the phylogenetic tree are shown in Figure 6B, and sequence alignment analysis revealed that BaMS05 had the highest similarity with *B. amyloliquefaciens* EB.DM3, reaching 98.72%. Further analysis of the phylogenetic tree indicated that BaMS05 clustered with starch-degrading *B. amyloliquefaciens* EB.DM3, suggesting that the isolated strain is a starch-degrading *Bacillus*.

### 3.6. Analysis of the Growth Characteristics of BaMS05

From the experimental results, it can be observed that the lag phase of BaMS05 is 0–3 h, entering the logarithmic growth phase before 3 h (Figure 8C). Subsequently, 30–36 h is the stationary phase of the bacterium, during which the proliferation rate of cells slows down, the number of deaths increases, the total number of bacteria reaches its maximum, and storage of substances such as glycogen and beta-hydroxybutyrate begins. Most *Bacillus* form spores at this stage. After 36 h, due to the accumulation of metabolic by-products and toxic substances in the environment, the cell death rate increases and cell morphology begins to deform, marking the decline phase.

The influence of temperature on the growth of BaMS05 (Figure 8C): The experimental results indicate that strain BaMS05 shows the optimal growth rate at 30 °C, reaching the highest OD600 value, indicating that this temperature is the most suitable growth temperature for BaMS05. At 40 °C, the growth rate of the strain is lower than at 30 °C, showing relatively good growth potential. In contrast, the growth rate of the strain at 20 °C and 50 °C is lower, exhibiting a slower proliferation trend. At 10 °C, the growth of BaMS05 is almost stagnant, with almost no change in OD600 value.

The influence of pH on the growth of BaMS05 (Figure 8C): Experimental results showed that BaMS05 could grow normally under initial pH conditions ranging from 6 to 9, reaching similar OD600 values after 24 h of cultivation, indicating that this pH range is suitable for the growth of BaMS05. However, under the initial pH condition of 5, the OD600 value remained almost unchanged, suggesting that this acidic environment has a significant inhibitory effect on the growth of BaMS05.

Effect of NaCl concentration on the growth of strain BaMS05 (Figure 8C): The strain showed optimal growth at a 0.5% NaCl concentration, with the highest OD600 value, indicating that this concentration is the most suitable salt concentration for the growth of BaMS05. With an increase in NaCl concentration to 5%, the growth of the strain was somewhat inhibited, but still able to grow. When the NaCl concentration reached 10% and 20%, the growth of the strain was significantly inhibited, especially at 20% concentration, where the strain almost did not grow. This indicates that high salt concentration has a significant inhibitory effect on the growth of the strain, likely related to increased osmotic pressure and cell membrane damage.

### 3.7. BaMS05 Has Inhibitory Effects on Various Pathogens of M. salmoides

**Figure 9:**
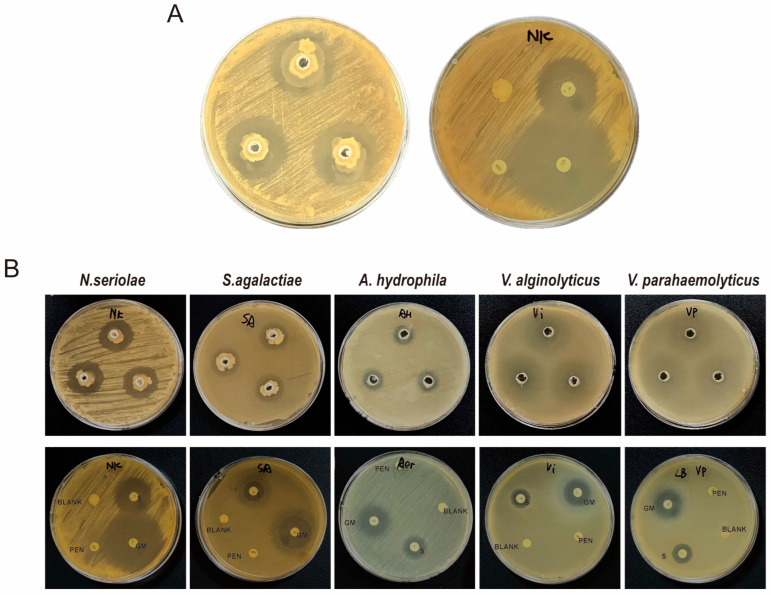
In vitro antagonistic activity of strain BaMS05 against *Nocardia* and other aquatic pathogens. (**A**) Antagonism against *N. seriolae*. (**B**) Inhibition spectrum showing zones of inhibition against *N. seriolae*, *S. agalactiae*, *A. hydrophila*, *V. alginolyticus*, and *V. parahaemolyticus*. Upper panels: antagonistic activity of BaMS05; lower panels: control assays with antibiotic discs (S: streptomycin, PEN: penicillin, GM: gentamicin) and blank discs.

The experimental results show (Figure 9) that strain BaMS05 can form obvious inhibition zones on the culture media of *N. seriolae*, *A. hydrophila*, *V. parahaemolyticus*, *V. alginolyticus*, and *S. agalactiae*, indicating that BaMS05 has significant antibacterial effects against these pathogenic bacteria. In addition, all five pathogenic bacteria showed resistance to penicillin, but no resistance to gentamicin and streptomycin.

### 3.8. BaMS05 Has the Ability to Colonize the Intestinal Tract of M. salmoides

**Figure 10:**
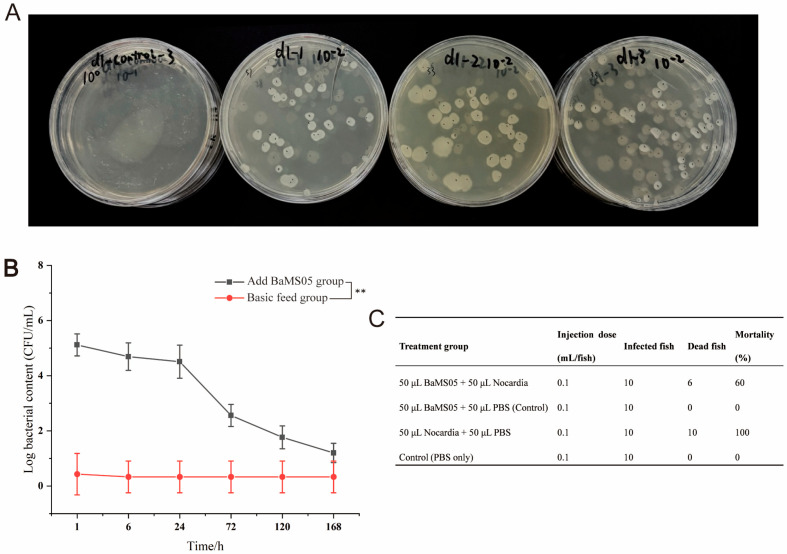
In vivo antagonistic activity of BaMS05 in *M. salmoides*. (**A**) Plate count analysis of intestinal homogenate dilutions of Day 1. (**B**) Colonization dynamics of BaMS05 in *M. salmoides* over 7 days (** indicates *p* < 0.01). (**C**) In vivo antagonistic efficacy against *Nocardia*.

In this study, two groups of experimental fish were fed with regular basic feed for *M. salmoides* and feed containing a concentration of 10^9^ CFU/g of BaMS05. The copy number of BaMS05 in their guts was counted within 7 d after feeding. The experimental results showed (Figure 10B) that on the first day after feeding the BaMS05-containing feed, the copy number of BaMS05 in the guts was about 10^5^ CFU/mL. By the third day, the BaMS05 had decreased to about 10^3^ CFU/mL. Subsequently, the number of BaMS05 in the guts of the experimental fish gradually decreased, and by the 7th day, the number of spore rods in the guts showed a significant difference to the control group. This result indicates that BaMS05 can colonize the guts of *M. salmoides* for at least 5 d.

### 3.9. BaMS05 Has a Certain Ability to Resist Infection by N. seriolae in M. salmoides

During the 14 d probiotics protection experiment, the experimental group of *M. salmoides* injected with the mixed BaMS05 showed a decrease in cumulative mortality to 60%, as compared to the control group with a cumulative mortality rate of 100%. This significant reduction in cumulative mortality demonstrates a clear protective effect (Figure 10C). The data suggest that BaMS05 may provide a certain degree of disease protection to *M. salmoides*.

## 4. Discussion

Currently, the *Nocardia* infection in aquaculture is quite challenging, often causing significant economic losses. Therefore, exploring methods for preventing and controlling *Nocardia* infection in fish is an important subject. Our finding is that *N. seriolae* infection reduces gut microbiota diversity in *M. salmoides*. This is consistent with previous studies, demonstrating the link between intestinal flora imbalance and susceptibility to disease in fish [20]. Furthermore, based on the observation of an increase in the proportion of *Bacillus* spores in *M. salmoides* that remained healthy after *N. seriolae* infection, we isolated, screened, and ultimately obtained a strain of starch-degrading BaMS05. This strain can inhibit the growth of *N. seriolae* and other common pathogens of *M. salmoides*, both in vivo and in vitro, and can colonize the gut of *M. salmoides*. This study not only deepens our understanding of the gut microbiota of *M. salmoides* and its relationship with *N. seriolae*, but also provides a potential new technical approach for the prevention and control of *N. seriolae* infection in *M. salmoides*.

*Nocardia* infection does not cause sepsis or acute immune reactions, but progressively invades various cells of fish, including white blood cells, with a long latency period. The infected fish are asymptomatic or have mild symptoms in the early stages of the disease, and by the time obvious symptoms or death are discovered, the infection has often been present for a long time, making treatment more difficult. This study found that *Nocardia* infection causes severe damage to important physiological and immune organs in fish, which is consistent with previous research results [21]. The important physiological and immune organs, such as the liver and kidneys of diseased fish, suffer severe damage, leading to decreased immune function. Moreover, *Nocardia* is a Gram-positive bacterium, and there are few drugs approved for use in aquaculture that are effective against Gram-positive bacteria in China. Besides, the susceptibility of *Nocardia* to antibacterial drugs is not well understood, and there is a lack of scientific basis for drug use. Due to various reasons, the treatment of *Nocardia* infection in fish is difficult, resulting in long duration, low cure rate, and high mortality rate [22]. Researchers are seeking alternative treatment methods for fish nocardiosis, such as vaccination, and it has been reported that researchers have developed an oral vaccine using probiotics recombinantly expressing the major antigen of *N. seriolae* in fish, which has shown good immune protection [23]. Yet, there are still no commercially available vaccines for *Nocardia* in fish. Even if commercialization of vaccines for *Nocardia* in fish is achieved in the future, there are still issues related to aquaculture costs as well as bacterial variations.

The gut microbiota of vertebrates is a complex microbial ecosystem, comprising diverse and abundant bacteria, archaea, and fungi. These gut microbial communities enhance metabolic capacity and provide a range of beneficial effects to their hosts, such as nutrient digestion, immune function, and resistance to invading pathogens. In the gut, there is a large population of symbiotic microorganisms that provide an excellent microecological environment for fish. In terms of nutrient digestion, the gut microbiota can produce vitamins, amino acids, digestive enzymes, various growth factors, and other metabolites. Major enzymes include carbohydrases, phosphatases, esterases, lipases, peptidases, cellulases, and proteases, which enzymes play a crucial role in the digestion of nutrients in the intestines [24]. Increasing research is focusing on the relationship between gut microbiota and the immune response and disease resistance of fish [25]. The interaction between the host and the gut microbial community is the basis for immune development. The genes of the microbiota or microbial cell populations exist within the organism and have unforeseen benefits and effects on overall health, from regulating the immune system to promoting growth and reproduction of the organism. The microbiota within the body have the ability to produce a variety of compounds through their genes, which have various benefits. Studies have shown that gut microbial communities can produce vitamins and help with food digestion, nutrient storage, and promote healthy metabolism. The microbiota also help maintain the integrity of the intestinal barrier cells, preventing harmful bacteria and toxins from penetrating the intestinal barrier, thus serving as an immune barrier [26].

Probiotics are defined as live microorganisms introduced into the gastrointestinal tract with food or water to promote health by enhancing the internal microbial balance. In recent years, probiotics have also been widely recognized as an environmentally friendly disease prevention method in aquaculture, especially in controlling bacterial fish diseases. The roles of probiotics in aquaculture include improving growth performance, disease resistance, immune enhancement, health status, balancing fish functional mechanisms, sustainability of intestinal microbiota, water quality (as bio-remediation to improve water quality), and enrichment of nutrients for zooplankton [27,28]. In this study, we focused on analyzing the antibacterial effect of BaMS05 and found that it inhibits the growth of various fish pathogenic bacteria, although its other effects are currently unclear. According to existing research, some *Bacillus* bacteria can inhibit the growth of pathogenic bacteria by producing antimicrobial peptides, biosurfactants, or competitive inhibition [29]. It is speculated that BaMS05 may also have a similar mechanism of action, such as directly inhibiting the growth of *N. seriolae* by secreting antimicrobial substances, or indirectly inhibiting its growth by competing for nutrients and attachment sites. However, these hypotheses require further experimental verification. In addition, for probiotics to function in fish, an important prerequisite is that they must be able to colonize the gut of fish. From this perspective, endogenous probiotics in the host may be more suitable in aquaculture, as selecting potential probiotics from the host or local environment can enhance the colonization ability in the intestines or improve the survival and growth of fry. Compared to exogenous probiotics, endogenous probiotics have several advantages, such as higher host safety, easier colonization and effectiveness, wider temperature and salinity adaptability, and stronger environmental adaptability. For example, Clements et al. found that the *Vibrio* bacteria mainly exists in the digestive tract of marine fish, especially in the hindgut of many herbivorous fish, providing the host with fatty acids and vitamins [30,31]. *Clostridium butyricum*, successfully used as a probiotic in aquaculture, enhances rainbow trout resistance to *Vibriosis*, stimulates immune responses, and improves the survival rate of Japanese flounder [32].

In the aquaculture industry, common probiotics used include *Bacillus*, lactic acid bacteria, butyric acid bacteria, and yeast. Lactic acid bacteria play an important role in maintaining the balance of the fish intestinal microbial community by inhibiting the reproduction of harmful bacteria through acidic metabolites or bacteriocins [33]. Butyric acid bacteria promote the proliferation of beneficial bacteria, inhibit the growth of pathogens, repair intestinal mucosa, reduce the occurrence of intestinal inflammation, and enhance the host’s immune function [34]. *Bacillus* is the most widely used probiotic in aquaculture because it can form spores resistant to environmental stress and adapt to harsh farming conditions. In addition, *Bacillus* produces various hydrolytic enzymes, such as proteases, amylases, cellulases, and lipases that help aquatic animals effectively utilize nutrients in feed; some *Bacillus* strains can also inhibit the growth of pathogens and enhance the immune response of aquatic animals, making them high-quality probiotic additives [35]. Although studies show that host-derived probiotics have advantages for host health, research on terrestrial animal gut microbes is more advanced and the characteristics of probiotics derived from terrestrial animals are more stable. On the other hand, the use of fish-derived probiotics in aquaculture is still in the early stages, so currently probiotics used in aquaculture mainly rely on probiotics derived from terrestrial animals, requiring further research and development. In this study, we obtained the starch-degrading *Bacillus* BaMS05, which can depress the proliferation of common pathogens in *M. salmoides* both in vivo and in vitro and can colonize the intestine of *M. salmoides*, showing potential in nocardiosis prevention and treatment in *M. salmoides*. Additionally, introducing BaMS05 into aquaculture systems may have an impact on the ecological environment. On the one hand, BaMS05 may improve the aquaculture environment by inhibiting the growth of pathogenic bacteria such as *N. seriolae*, thereby promoting the healthy growth of fish [36]. On the other hand, the introduction of BaMS05 may also alter the existing microbial community structure in the water body, potentially affecting other beneficial microorganisms. Therefore, future research should further explore the mechanism of action of BaMS05, assess its long-term ecological impacts in aquaculture systems, and formulate reasonable application strategies to ensure its safety and efficacy.

## 5. Conclusions

In summary, this study found that infection with *N. seriolae* significantly altered the intestinal microbiota structure of *M. salmoides*, with a decrease in the diversity of the microbiota and a change in the dominant microbiota during the acute phase, and a gradual recovery during the stabilization phase. A strain of *Bacillus amyloliquefaciens* MS05 was screened for its significant antibacterial effect against *Nocardia* and common aquatic pathogens. The strain not only colonized the intestinal tract of *M. salmoides*, but also significantly reduced the mortality rate caused by *N. seriolae* in infected fish. In the following research, the biosafety of BaMS05 will be further verified, and its fermentation process and usage methods will be optimized, providing a theoretical and experimental basis for its application in preventing and controlling *Nocardia* infection in *M. salmoides*.

## Figures and Tables

**Table 1:** PCR specific primers.

Primers	Sequence (5′-3′)	Length (bp)
NK-659-F	ATCTGGAGCCGATTCGGACTG	566
NK-659-R	ACGCCACTGATCACTCGCATTT	

**Table 2:** Results of physiological and biochemical assays for the bacterial strain.

Item	Result
Spore-forming	+
Growth at pH 5.7	+
Nitrate reduction	+
Propionate utilization	−
Tolerance to 7% NaCl	+
Starch hydrolysis	+
D-Xylose fermentation	+
L-Arabinose fermentation	+
Voges–Proskauer(VP) test	+
Citrate utilization	+
D-Mannitol fermentation	−
Gelatin liquefaction	+

Note: “+” indicates a positive result (capable of reaction/growth); “−” indicates a negative result (no reaction/growth).

## Data Availability

Data will be made available upon request.

## Supplementary Materials

The following supporting information can be downloaded at: https://www.mdpi.com/article/10.3390/biology14091128/s1. Figure S1: Principal Component Analysis plot (PCA). The x-axis represents the first principal component (PCA1), and the percentage indicates the contribution of the first principal component to the variation among samples. The y-axis represents the second principal component (PCA2), and the percentage indicates the contribution of the second principal component to the variation among samples. Figure S2: Microbial biomarker analysis in *M. salmoides* using LDA. Bar plot of LDA scores showing microbial biomarkers with significant intergroup differences (LDA score > 3.0, default threshold). The length of each bar represents the effect size of differential taxa (LDA score). Table S1: Data and quality control processing statistics table.
