# Supplementary material for: Study on the Nocardia seriolae Antagonistic Bacterium in the Gut Microbiota of Micropterus salmoides
Source: Biology (Basel). 2025 Aug 26;14(9):1128. doi: 10.3390/biology14091128 (PMC12467488; doi:10.3390/biology14091128)
Supplement: Supplementary file 1 [file biology-14-01128-s001.zip › Original Images for Gels.pdf]

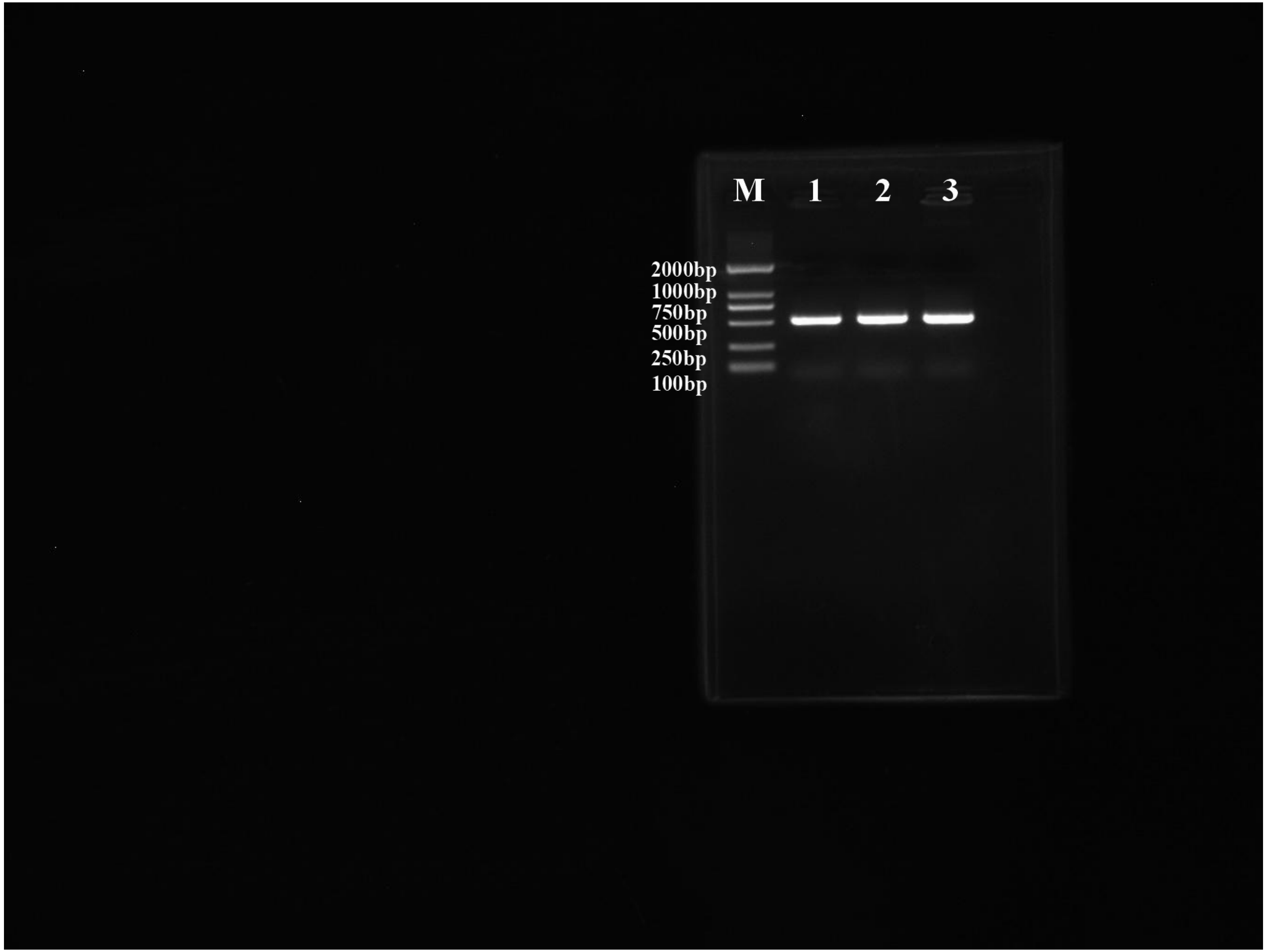

Partial sequence amplification of *M. salmoides* liver.

1:liver PCR products-1; 2:liver PCR products-2; 3:liver PCR products-3
