# Supplementary material for: Study on the Nocardia seriolae Antagonistic Bacterium in the Gut Microbiota of Micropterus salmoides
Source: Biology (Basel). 2025 Aug 26;14(9):1128. doi: 10.3390/biology14091128 (PMC12467488; doi:10.3390/biology14091128)
Supplement: Supplementary file 1 [file biology-14-01128-s001.zip › Supplementary Figure.pdf]

Supplementary Figure

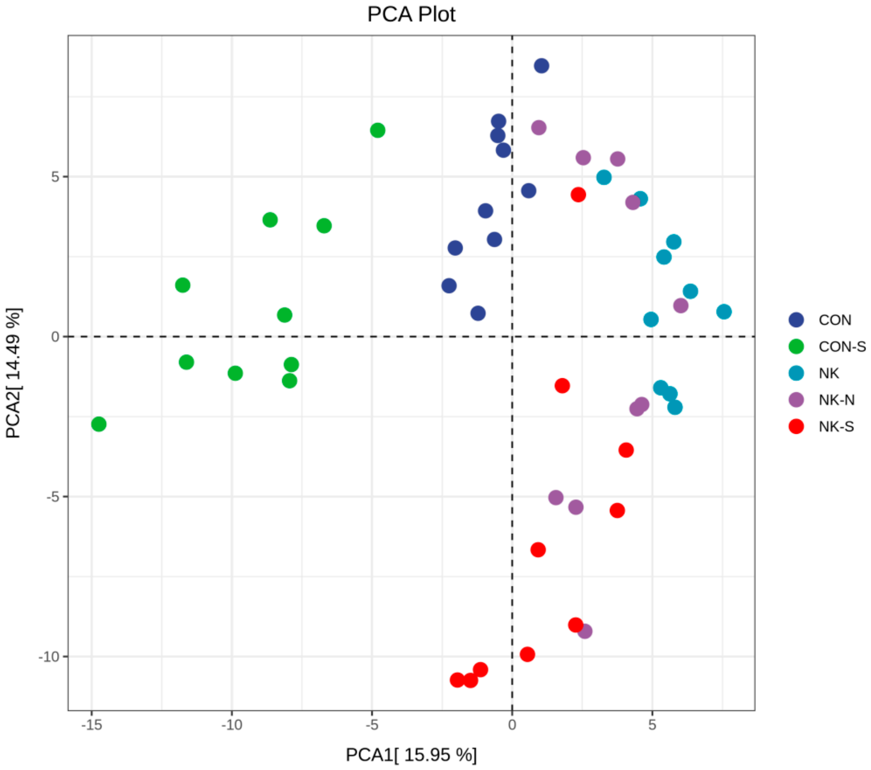

Figure S1. Principal Component Analysis plot (PCA). The x-axis represents the first principal component (PCA1), and the percentage indicates the contribution of the first principal component to the variation among samples. The y-axis represents the second principal component (PCA2), and the percentage indicates the contribution of the second principal component to the variation among samples.

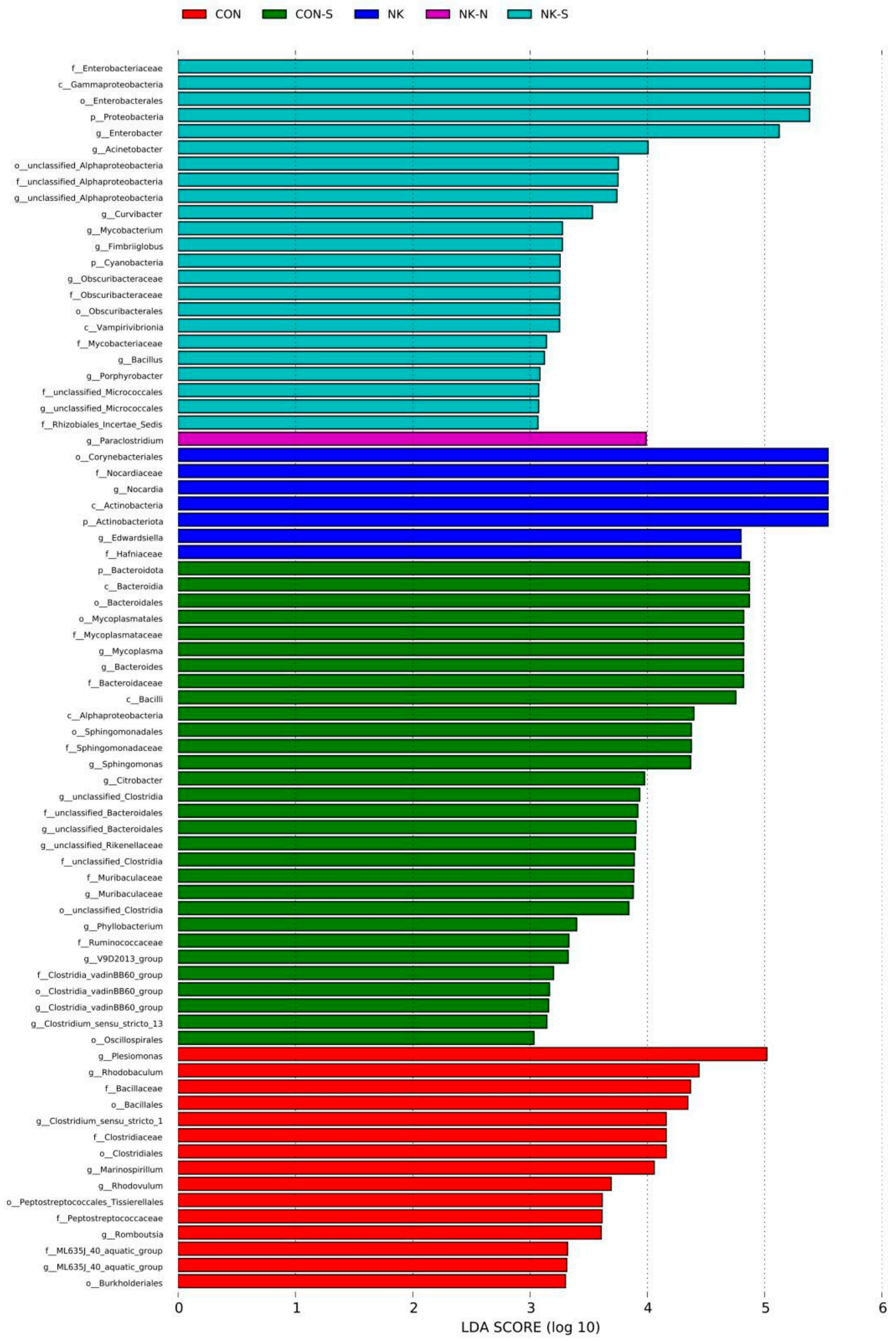

Figure S2. Microbial biomarker analysis in *M. salmoides* using LDA. Bar plot of LDA scores showing microbial biomarkers with significant intergroup differences (LDA score > 3.0, default threshold). The length of each bar represents the effect size of differential taxa (LDA score).
